# Federal Advisory Councils Established to Assist Working Caregiving Families

**DOI:** 10.1093/geroni/igaa057.2555

**Published:** 2020-12-16

**Authors:** Ethlyn McQueen Gibson

**Affiliations:** Hampton University, Hampton, Virginia, United States

## Abstract

This presentation will describe the legislative charge of two advisory councils established in 2018 through the Recognize, Assist, Include, Support and Engage Family Caregivers Act and the Supporting Grandparents Raising Grandchildren Act to support working family caregivers. The advisory councils are charged with 1) providing recommendations on effective models of family caregiving 2) improving coordination across federal government programs; and 3) identifying, coordinating, and promoting information, resources, and 4) best practices for working grandparents raising grandchildren, while maintaining their own physical, mental, and emotional well-being. The experiences of working family caregivers will be the foundation for these recommendations that are being developed over the next two years to present to Congress. These recommendations will support the development and execution of a national family caregiving strategy.

